# Accuracy and Bias of Pulse Oximetry in the Intensive Care Unit: A Prospective Observational Study

**DOI:** 10.1111/nicc.70433

**Published:** 2026-03-19

**Authors:** Kaio B. Barros, Jocassia S. Pinheiro, Murilo S. Sampaio, Luismar B. Cruz Junior, Fernando F. Ferreira, Anibal Basile‐Filho, Rinaldo R. J. Guirro, Luciano Bachmann

**Affiliations:** ^1^ Department of Physics University of São Paulo Ribeirão Preto Brazil; ^2^ Department of Health Science University of São Paulo Ribeirão Preto Brazil; ^3^ Sao Carlos Institute of Physics University of São Paulo São Carlos Brazil; ^4^ Department of Surgery and Anatomy University of São Paulo Ribeirão Preto Brazil

**Keywords:** oxygen saturation, pulse oximetry, racial bias, skin colorimetry

## Abstract

**Background:**

Non‐invasive oximetry is crucial for the easy and continuous monitoring of blood oxygenation, from routine assessments to critical care in intensive care units (ICU). Studies indicate that pulse oximeter readings may be biased due to the high light absorption of melanin.

**Aim:**

This study aims to analyse the agreement among three different pulse oximeters and their concordance with arterial blood gas measurements in ICU patients and verify how accuracy varies with age and skin pigmentation.

**Study Design:**

We conducted a prospective cross‐sectional observational study with a population of intensive care patients. Blood oxygen saturation was measured simultaneously by pulse oximetry and arterial blood gas analysis. Skin pigmentation was objectively assessed at two anatomical sites using calibrated colorimeters and quantified by the individual typology angle (ITA°).

**Results:**

Among 100 ICU patients (SaO_2_ 85%–100%), two of the three oximeters exceeded the 3% accuracy root mean square (ARMS) 2013 FDA threshold, whereas one device remained within limits (2.91%). Agreement with arterial oxygen saturation was weak (CCC = 0.34–0.46) and lower SaO_2_ was associated with overestimation increase (0.31%–0.54% for every 1% SaO_2_ decrease). Multivariable models showed no evidence of association between ITA° and SpO_2_ error (*p* > 0.05). In contrast, older age predicted greater overestimation (*β* range = 0.05%–0.06% per year; 95% CI range = 0.02–0.09; all *p* ≤ 0.05).

**Conclusions:**

Although estimates suggested small pigmentation effects, analysis indicated these were indistinguishable from random error. In contrast, physiological factors such as lower SaO_2_ and older age were consistently associated with greater oximeter error. Substantial clinical variability limits detection of small biases, underscoring the need for improved calibration and validation using objective skin‐tone assessment.

**Relevance to Clinical Practice:**

In intensive care, where continuous monitoring is essential, pulse oximeter error was more strongly related to physiological factors, such as arterial oxygen saturation and age, than to skin tone. These findings support cautious interpretation of SpO_2_ values and appropriate device selection to ensure accurate assessment across diverse populations.

## Impact Statements

- What is known about the topic?
  ○ Pulse oximetry is an essential tool of non‐invasive monitoring in critical care, yet accuracy can be compromised by device design and patient factors.
  ○ Melanin absorption has been implicated in measurement bias, but a lot of studies have relied on self‐reported race rather than objective pigment metrics.
  ○ Changes in perfusion and haemodynamics may further degrade signal quality, potentially confounding oximetry readings in ICU patients.
- What this paper adds
  ○ Commercial pulse oximeters commonly used in home and clinical settings underperformed against regulatory ARMS thresholds in ICU patients, emphasising the need for rigorous device validation and informed selection.
  ○ The study provides new evidence on skin tone‐related bias in pulse oximetry across a diverse patient population, highlighting equity considerations in device performance.
  ○ Patient age consistently predicts SpO_2_–SaO_2_ discrepancy, underscoring the need to account for perfusion‐related confounders in clinical interpretation.

## 1 Introduction and Background

Understanding the interaction between light and human skin is crucial in many areas of medical technology, including diagnostics and therapeutic applications [1]. The transmission of light through the skin is strongly influenced by its optical properties, primarily the absorption and scattering coefficients [2]. These coefficients are determined by the skin composition and directly depend on the concentration of various chromophores, including melanin, haemoglobin and water. These chromophores affect light interaction in complex ways, and variations in their concentration can influence the outcomes of non‐invasive optical techniques. As melanin plays a key role in defining skin colour, differences in melanin levels significantly affect the depth of light penetration across varying skin tones [3, 4, 5].

Pulse oximetry is a widely used, non‐invasive method for assessing blood oxygen saturation. It utilises two light‐emitting diodes (LEDs) at red (~660 nm) and infrared (~940 nm) wavelengths, both within the ‘optical window’ (600–1100 nm), a spectral range where biological tissues allow deeper light penetration [6]. Pulse oximeters estimate arterial haemoglobin oxygen saturation (SaO_2_) by analysing the ratio of pulsatile to total transmitted red light and comparing it to the same ratio for infrared light as it passes through a fingertip, earlobe, or other tissue [7]. While haemoglobin is the primary absorber of these wavelengths, melanin can also absorb light in this range, potentially affecting measurement accuracy in individuals with higher melanin concentrations [8].

Studies have suggested that pulse oximetry may exhibit a racial bias, potentially delivering less accurate readings for individuals with darker skin, and in states of low oxygenation [9, 10, 11, 12, 13, 14, 15, 16, 17, 18, 19, 20, 21, 22, 23, 24, 25, 26]. One of the most significant limitations in most studies is the lack of objective measurement for skin tone, as it was either not assessed or was evaluated subjectively [27, 28, 29, 30, 31, 32]. This finding has raised concerns, highlighting the need to investigate how skin colour may impact oximetry accuracy and the broader implications for patient care, as it could influence critical decisions related to fluid management and ICU admissions [32, 33, 34, 35, 36, 37].

## 2 Aims

In this study, we aim to investigate this issue within the Brazilian population, which is characterised by a diverse range of skin tones [38]. By comparing pulse oximetry readings with arterial blood gas analysis (ABG) as a reference and utilising colorimetry for objective skin tone measurement, we seek to determine whether skin tone affects the reliability of pulse oximetry. This approach will yield insights into the potential limitations of oximetry across varied skin tones and support the development of more inclusive medical technologies.

## 3 Design and Methods

### 3.1 Setting and Sample

This prospective cross‐sectional study, reported in accordance with GRRAS (Guidelines for Reporting Reliability and Agreement Studies), was conducted in the adult intensive care unit (ICU) of a tertiary‐care hospital to compare arterial oxygen saturation (SaO_2_, gold standard by blood gas analysis) with peripheral oxygen saturation (SpO_2_) measured by three pulse oximeter models.

We included patients aged ≥ 18 years who were admitted to the ICU and performed ABG in 2024 by normal medical prescription. We excluded patients that could not realise finger oximetry due to physical impediment, bandage dressing or poor peripheral perfusion. Sample size calculations were performed using ENE v3.0 (Universidad Autónoma de Barcelona, Spain), based on the means and standard deviation reported by Pilcher et al. [39], with assumed SpO_2_–SaO_2_ bias means of 0.1, −1.1 and −1.2 percentage points and a standard deviation of 1.7 percentage points. The calculation was based on a paired comparison of means. To achieve 80% power at a two‐sided *α* = 0.05 and allow for a 15% attrition rate, approximately 100 paired measurements per device were required. The baseline demographics are presented in Table 1 (located in the Results Section).

**TABLE 1:** Baseline demographics and ITA° skin pigmentation distribution.

	Baseline characteristics
Age (years), mean ± SD [min, max]	58 ± 16 [18, 90]
Sex *n* (%)	
Male	54 (54%)
Female	46 (46%)
Self‐reported ethnicity *n* (%)	
White	79 (79%)
Brown	15 (15%)
Black	6 (6%)
ITA° dorsal hand skin, mean ± SD [min, max]	2.67 ± 25.21 [−73.01, 54.02]
ITA° palmar distal phalanx skin, mean ± SD [min, max]	28.59 ± 18.52 [−26.21, 67.18]
ITA° dorsal hand skin subgroup *n* (%)	
1—very light	0 (0%)
2—light	6 (6%)
3—intermediate	11 (11%)
4—tan	18 (18%)
5—brown	55 (55%)
6—dark	10 (10%)

*Note:* Continuous variables are presented as mean ± standard deviation; values in square brackets represent minimum and maximum. Categorical variables are presented as *n* (%). Sex reported as recorded in medical records; gender identity was not assessed. ITA° subgroups are presented for descriptive purposes only and were not used for stratified or comparative analyses.

Abbreviation: ITA°, individual typology angle.

### 3.2 Data Collection Tools and Methods

Patients had their oxygen saturation levels measured using both pulse oximetry and arterial blood gas analysis concurrently. Three different ANVISA/FDA approved oximeters were employed for the measurements: finger oximeters G‐Tech Oled graph (G‐Tech Optoelectronics Corporation, Miaoli, Taiwan) and Multilaser HC023 (Multilaser, São Paulo, Brazil) and the pulse oximeter Nihon Kohden Life Scope G5 CSM‐15000 (Nihon Kohden Corporation, Tokyo, Japan), which is already used at the hospital. The ABG was performed by the nurses under normal circumstances and analysed by potentiometry and amperometry under 37°C. The data collected via oximetry were separated by the employed model and were processed to be compared with the arterial blood gas measurements, which provided the gold standard for real oxygenation.

To study the influence of skin pigmentation on the accuracy of pulse oximetry, colorimetry was performed on two regions of each participant: the dorsum of the hand and the palmar surface of the finger used for oximetry. This approach allowed for an assessment of individual colour and melanin concentration in the area where the oximeter light interacts. The device employed was the colorimeter/spectrophotometer Delta Vista 450G (Delta Colour, São Leopoldo, Rio Grande do Sul, Brazil) with BCRA Series II calibration certificate and aperture size of 4 mm; the measures were made in triplicate. The colorimetry gives three components in the L*a*b* colour space, where L* corresponds to the luminosity [0–100], a* to the green and red tones [−50 to 50] and b* to blue and yellow tones [−50 to 50].

To characterise the skin shades, we utilised the Individual Typology Angle (ITA°) that simplifies the colour components into a single parameter and is calculated using Equation (1) [40].

(1) ITA°=180πtan−1L*−50b*

Additionally, aiming to facilitate the sample description, we separate subjects by subgroups using the ITA° as follows: higher than 55° = very light, 55° to 41° = light, 41° to 28° = intermediate, 28° to 10° = tan, 10° to −30° = brown and lower than −30° = dark.

Before and after each measurement, the region and the equipment were cleaned and dried to prevent cross‐contamination and ensure greater accuracy and reliability of the results. Aseptic procedures followed the standards established by the healthcare unit.

### 3.3 Data Analysis

For each oximeter, we computed mean bias (SaO_2_–SpO_2_), standard deviation of differences and analytic 95% limits of agreement (bias ±1.96 SD) with confidence intervals via the methods of Bland & Altman and Ludbrook. Accuracy root mean square (ARMS) error was reported as a point estimate. Concordance correlation coefficient (CCC) and its 95% CI were derived using a Fisher *z*' transform approach. Proportional and fixed bias were modelled via Deming regression (SciPy ODR), treating SaO_2_ as the independent variable and SpO_2_ as the dependent variable. This method accounts for measurement error in both axes and yields more reliable estimates of systematic and proportional effects. Additionally, multivariable ordinary least‐squares regression was performed with bias (SpO_2_–SaO_2_) as the dependent variable and ITA° (finger, hand) and age as predictors to explore covariate effects and potential confounders. All multivariable models were additionally adjusted for SaO_2_ to control confounding by the true oxygenation level. Prior to model construction, the relationship between dorsal and palmar ITA° measurements was assessed using Pearson correlation. Multicollinearity was formally evaluated using variance inflation factors (VIFs), with values < 5 considered indicative of negligible collinearity. Both variables were retained in the models when these assumptions were met. Heteroscedasticity was assessed using Breusch‐Pagan test and regression inferences were reported using heteroscedasticity‐consistent (HC3) standard errors. Differential bias confidence intervals were computed via non‐parametric bootstrap (resampling participants with replacement; 5000 replications) to obtain robust empirical 95% CIs. All analyses were implemented in Python 3.10 with pandas, statsmodels, SciPy and pingouin. The study was designed and reported in accordance with the Guidelines for Reporting Reliability and Agreement Studies (GRRAS) [41], and the checklist is provided as Supporting Information.

### 3.4 Ethical and Institutional Approvals

Ethical approval was obtained in 12/2023 from the Institutional Research Ethics Committee (CEP) under CAAE 74692423.7.0000.5407 and all participants (or their legal surrogates) provided written informed consent prior to enrolment.

## 4 Results

**FIGURE 1:**
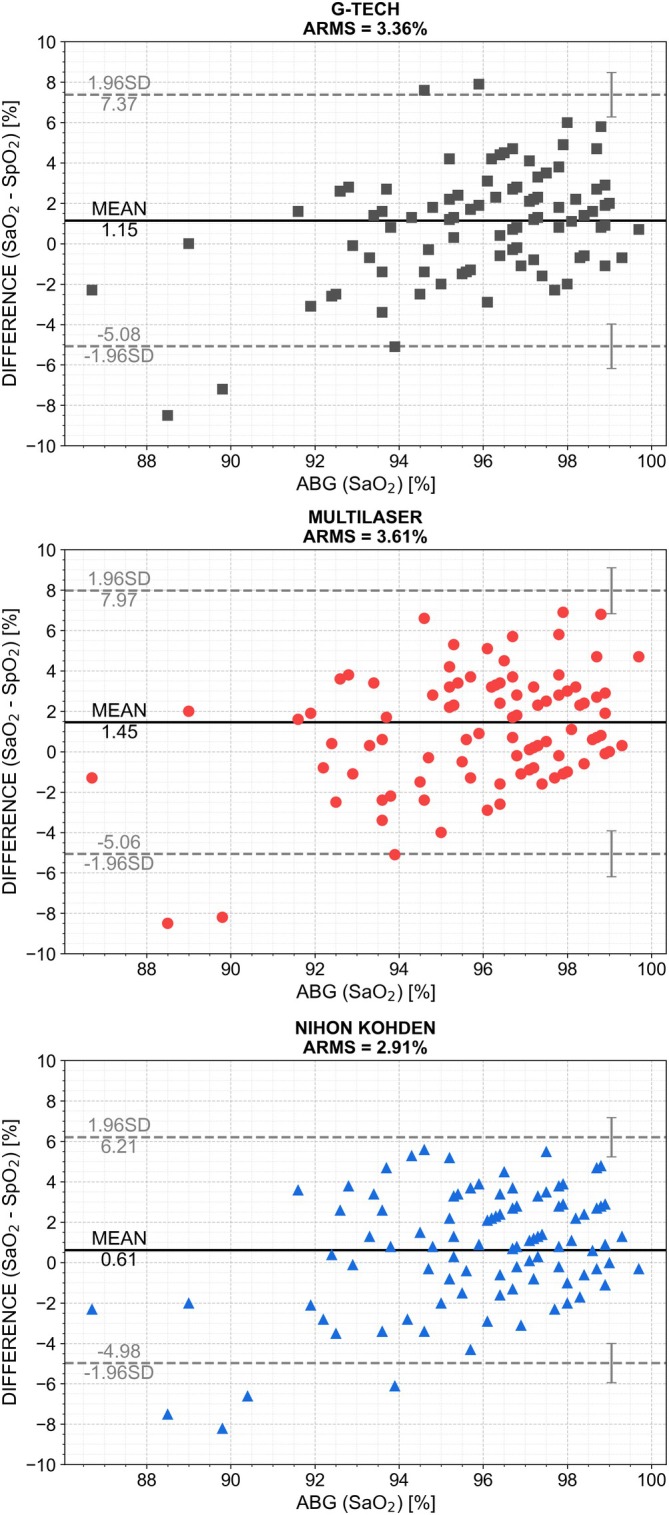
Bland–Altman plots comparing SaO_2_ (ABG, gold standard) with SpO_2_ readings from the oximeters (G‐Tech, Multilaser and Nihon Kohden, respectively). Markers represent paired measurements; solid black lines indicate mean bias; dashed grey lines show 95% limits of agreement (±1.96 SD).

In total, 100 patients were included, and the statistical analysis for each oximeter in the range of 86%–100% SaO_2_ is displayed in Table 2 and shows that 2 of the 3 do not reach the ARMS < 3% 2013 FDA guidance [42]. Specifically, the ARMS values were (3.36%) for G‐Tech, (3.61%) for Multilaser and (2.91%) for Nihon Kohden. The mean bias was smallest for Nihon Kohden (0.61%; 95% CI 0.05%–1.18%), suggesting that its SpO_2_ readings were, on average, closer to the arterial blood gas reference, whereas larger mean biases were observed for G‐Tech (1.15%; 95% CI 0.5%–1.79%) and Multilaser (1.45%; 95% CI 0.79%–2.12%). Consistently, Nihon Kohden also exhibited narrower limits of agreement (−4.98% to 6.21%; 95% CI, −5.95% to 7.18%) compared with G‐Tech (−5.08% to 7.37%) and Multilaser (−5.06% to 7.97%), indicating reduced random error and improved precision relative to the other devices. These patterns are visually corroborated by the Bland–Altman plots presented in Figure 1.

**TABLE 2:** Device accuracy: ARMS, bias, LoA, CCC and Deming regression results.

	G‐Tech (*n* = 96)	Multilaser (*n* = 99)	Nihon Kohden (*n* = 100)
Wavelength (nm)	670, 905	670, 900	660, 940
ARMS (%)	3.36	3.61	2.91
Mean bias (%) ± SD (95% CI)	1.15 ± 3.18 (0.50, 1.79)	1.45 ± 3.33 (0.79, 2.12)	0.61 ± 2.85 (0.05, 1.18)
Upper LoA (95% CI)	7.37 (6.27, 8.47)	7.97 (6.83, 9.11)	6.21 (5.24, 7.18)
Lower LoA (95% CI)	−5.08 (−6.18, −3.98)	−5.06 (−6.20, −3.93)	−4.98 (−5.95, −4.01)
CCC (95% CI)	0.34 (0.15, 0.50)	0.37 (0.19, 0.53)	0.46 (0.29, 0.64)
Deming regression			
Slope	0.55	0.46	0.69
Intercept	44.04	52.36	30.02
*R* ^2^	0.14	0.20	0.23

Abbreviations: ARMS, accuracy root mean square; CCC, Lin's concordance correlation coefficient; CI, confidence interval; LoA, limits of agreement (Bland–Altman).

All three oximeters demonstrated low concordance with arterial oxygen saturation, with Lin's concordance correlation coefficients (CCC) of 0.46 (95% CI 0.29–0.64) for Nihon Kohden, 0.34 (95% CI 0.15–0.50) for G‐Tech and 0.37 (95% CI 0.19–0.53) for Multilaser, indicating substantial discrepancies between SpO_2_ and SaO_2_ measurements. Even the best‐performing device, Nihon Kohden, achieved at most moderate agreement, remaining well below levels generally considered acceptable for clinical decision‐making. In contrast, Multilaser and G‐Tech showed wider limits of agreement and lower CCC values, indicating greater dispersion and variability in measurement error.

Deming regression analysis revealed slopes below unity for all oximeters, indicating proportional bias, with estimated slopes of 0.55 for G‐Tech, 0.46 for Multilaser and 0.69 for Nihon Kohden. Nihon Kohden's slope was closest to 1, suggesting that its measurement error scaled less with true saturation level. These slopes translate clinically: for every 1% decrease in SaO_2_, the bias (SaO_2_–SpO_2_) increased by 0.31%–0.54% across devices (calculated as 1 − slope). Intercepts were highest for G‐Tech (44.04), consistent with a larger fixed bias, whereas Nihon Kohden exhibited a smaller intercept (52.36), indicating fewer systematic discrepancies, particularly at lower saturation values. The coefficients of determination (*R* ^2^) were low across all devices (0.14–0.23), indicating weak linear association between SpO_2_ and SaO_2_.

Consistent with these findings, all devices demonstrated less satisfactory results at lower blood gas saturation levels, which is clinically concerning given the increased need for monitoring and oxygen supplementation in hypoxemic patients. Some patients did not exhibit results for certain devices, which explains the smaller sample size (*n*) for some oximeters.

To evaluate the relationship between measurement error and skin pigmentation, we used the ITA° scale and quantified skin‐tone bias using two complementary approaches. First, we applied the differential bias method proposed by the FDA. By fitting a linear regression and estimating the difference between the bias at ITA −50° and ITA 50° for dorsal hand skin and ITA 0° and ITA 60° for palmar DP skin, we can determine the skin‐tone bias or differential bias (DB) for each device and measure site. The FDA suggests (not require) that the absolute differential bias (ADB) in the range of 85%–100% SaO_2_ (employed here) may not exceed 2.0% [42]. Therefore, we found a DB on dorsal hand skin of −2.05% (95% CI −4.99, 0.87), −2.04% (95% CI −4.78, 0.83), 0.35% (95% CI −1.86, 2.81), for G‐Tech, Multilaser and Nihon Kohden, respectively. On the palmar DP skin, DB values were 1.15% (95% CI −0.66, 2.83), 0.82% (95% CI −1.40, 3.10), 1.29% (95% CI −0.32, 2.72), for G‐Tech, Multilaser and Nihon Kohden, respectively. Bootstrap 95% CIs for all devices crossed zero, indicating that none of these slopes were statistically distinguishable from no skin‐tone effect.

In addition to the FDA‐proposed differential‐bias method, we fitted multivariable ordinary least‐squares (OLS) regression models to evaluate the association between SpO_2_–SaO_2_ discrepancy and patient characteristics. Table 3 summarises these models, which included skin pigmentation (continuous ITA°) and age as predictors. The intercept is omitted from the table because it has no clinical interpretation (it corresponds to implausible covariate values). Correlation between dorsal and palmar ITA° was weak (Pearson *r* = 0.23; *R* ^2^ ≈ 0.05). Consistently, multicollinearity diagnostics showed low variance inflation factors across all device‐specific models (VIF range: 1.06–1.16), indicating that both variables could be jointly included without compromising model stability. Across all three devices, age emerged as the only consistent and significant predictor, with positive regression coefficients (*β* range = 0.05–0.06 per year, CI range = 0.02–0.09; all *p* ≤ 0.05) indicating a greater tendency for the oximeters to overestimate saturation in older patients. In contrast, no evidence of an association between skin pigmentation (dorsal hand or palmar DP ITA°) and SpO_2_–SaO_2_ discrepancy was observed in any of the models, with *p* values consistently above 0.05.

**TABLE 3:** Multivariable linear regression of SpO_2_–SaO_2_ bias: effects of ITA and age.

Predictor	*β* coefficient	Std. error	*t* value	Pr(>|*t*|)	CI 95%
G‐Tech					
ITA° dorsal hand (per 1°)	−0.02	0.01	−1.33	0.18	(−0.05, 0.01)
ITA° palmar DP (per 1°)	0.00	0.02	0.10	0.92	(−0.04, 0.04)
**Age (per year)**	**0.05**	**0.02**	**3.39**	**0.01**	**(0.02, 0.09)**
Multilaser					
ITA° dorsal hand (per 1°)	−0.02	0.02	−1.16	0.24	(−0.06, 0.01)
ITA° palmar DP (per 1°)	0.00	0.03	0.06	0.95	(−0.05, 0.05)
**Age (per year)**	**0.05**	**0.02**	**2.79**	**0.05**	**(0.02, 0.09)**
Nihon Kohden					
ITA° dorsal hand (per 1°)	0.00	0.01	0.26	0.80	(−0.02, 0.03)
ITA palmar DP (per 1°)	0.00	0.02	−0.04	0.97	(−0.03, 0.03)
**Age (per year)**	**0.06**	**0.02**	**3.59**	**< 0.01**	**(0.03, 0.09)**

*Note:* Multivariable linear regression models were fitted separately for each device, with SpO_2_–SaO_2_ bias (%) as the dependent variable. *β* coefficients represent the expected change in bias per unit increase in each predictor. ITA° was modelled as a continuous variable (per 1‐degree increase), and age was modelled per 1‐year increase. Statistically significant predictors (*p* ≤ 0.05) are shown in bold.

Abbreviations: CI, confidence interval; DP, distal phalanx.

## 5 Discussion

Our study found no evidence of skin‐tone bias in oximetry measurements, contrasting with findings from other publications. This discrepancy may reflect key methodological differences. Many prior studies rely on self‐reported race or ethnicity, which introduces subjectivity and misclassification [7, 9, 24, 26, 35]. Table 1 demonstrates these limitations, showing substantial inconsistencies and overlap between self‐identified racial categories and objectively measured skin tone using ITA°. These findings indicate that self‐reported race does not accurately represent actual skin pigmentation, limiting the reliability of studies based solely on subjective classification. The Fitzpatrick scale was not used because it reflects sunburn response rather than true skin colour, unlike ITA°, which directly corresponds to melanin concentration and optical absorption [5]. To address these limitations, we used objective colorimetry to quantify skin pigmentation independently of self‐identification, ensuring precise measurement. This methodological approach strengthens the validity of our findings and highlights the importance of objective skin‐tone assessment when evaluating oximetry accuracy, contributing to more robust and equitable medical evaluation.

Additionally, multicentre studies that pool oximetry and ABG data from different hospitals can introduce bias because facilities vary in resources, staff training and patient acuity [23]. Patients at under‐resourced hospitals often have worse perfusion and more severe illness, which degrades SpO_2_ accuracy; if these hospitals serve socio‐economically disadvantaged and frequently racially minoritised populations, the resulting measurement errors may falsely appear as ‘racial bias’ [24, 26, 32]. By conducting a single‐centre study with standardised equipment and protocols, we minimise these inter‐hospital and socio‐economic confounders and focus on true device and physiologic effects. For nurses, this highlights that oximeter performance may vary by hospital resources and equipment. When using different devices or caring for transferred patients, awareness of potential variability is warranted.

Another factor that may explain the absence of detectable racial bias in our dataset relates to the statistical rigour applied in estimating uncertainty. Whereas FDA guidance relies primarily on point estimates derived from linear fits of bias across pigmentation levels, such estimates do not distinguish true systematic bias from random measurement variability. Our use of confidence intervals, bootstrap resampling and model‐based inference accounts for sampling variability and demonstrates that a point estimate alone cannot confirm or exclude bias; it may fall within expected random error. Importantly, real‐world oximetry performance often exhibits substantial variance, particularly in lower‐cost devices and hospital environments, and this imprecision can obscure small systematic differences. In critical care practice, this highlights that overall device variability may represent a more immediate concern for nurses than small, statistically uncertain bias estimates, particularly when titrating oxygen therapy based on continuous SpO_2_ monitoring.

In contrast to skin pigment, age emerged as a predictor of SpO_2_–SaO_2_ error across all devices, with older patients exhibiting larger positive differences (overestimation by oximeters). This age effect may reflect confounding by overall health status, since elderly ICU patients often have poorer peripheral perfusion, hypotension or vasoconstriction, all of which degrade photoplethysmographic signal quality and broaden measurement error. In critically ill populations, diminished cardiac output and altered haemodynamics further attenuate waveform amplitude, exacerbating age‐related bias. In such situations, nurses should interpret SpO_2_ values with greater caution, recognising a broader margin of uncertainty and considering arterial blood gas confirmation when clinical decisions depend on precise assessment of oxygenation.

## 6 Limitations

A limitation of this study is the small number of data points with arterial oxygen saturation below 88% and none below 85%. This limited range prevents an evaluation of oximeter performance at lower saturation levels, where both larger measurement errors and potential skin‐tone biases might appear or become more pronounced. In addition, the study did not systematically collect other physiological variables known to influence pulse oximetry accuracy, such as body temperature or hydration status, which may have contributed to residual confounding. Furthermore, our sample lacked representation from individuals classified in ITA° subgroup 1, corresponding to the lightest skin tones. This may be partially explained by the use of the dorsal hand site for colorimetry, a region typically more exposed to sunlight and also reflect the predominant skin phototypes commonly observed in the local population. Additionally, another limitation of this study is that it was conducted in a single ICU at one tertiary‐care hospital, which may restrict the generalisability of the findings to other clinical settings or countries. Nevertheless, the single‐centre design ensured standardised equipment, trained staff and consistent protocols.

## 7 Implications for Practice and Further Research

Clinical teams must be aware that consumer‐grade pulse oximeters may not meet ARMS < 3% thresholds in critically ill patients and should preferentially deploy devices demonstrated to perform reliably in the ICU. Regular calibration and performance checks against arterial blood gas measurements can help identify and mitigate device inaccuracies. Training on the limitations of pulse oximetry, emphasising risk factors and objective pigment metrics, will empower nurses and clinicians to interpret SpO_2_ readings more safely and effectively.

Future research should prioritise the development and testing of pulse oximeter designs with optimised LED wavelengths and sensor geometries to enhance signal separation and minimise bias across skin tones. Large‐scale, multicentre studies are needed to evaluate these innovations in diverse ICU populations, including oxygen saturation levels below 85%. Collaborative efforts between clinicians, engineers and regulatory agencies will be essential to translate these findings into new devices and evidence‐based practice guidelines.

## 8 Conclusions

Our findings indicate that two of the three oximeters tested did not meet 2013 FDA guidances, raising concerns about their reliability in clinical scenarios. Although linear models suggested small differences related to skin pigmentation, the incorporation of bootstrap resampling and confidence intervals showed that these estimates were highly uncertain. Our data therefore do not demonstrate skin‐tone bias evidence, although a small effect cannot be entirely excluded.

In contrast, arterial oxygen saturation and age showed consistent and strong associations with measurement error across all devices. Lower SaO_2_ values and older age were linked with greater overestimation, aligning with expected physiological vulnerabilities in critically ill patients. These factors appear to play a more important role in oximetry inaccuracy than skin pigmentation.

Overall, the main concern identified is the magnitude of random error observed in routinely used pulse oximeters, which limits measurement reliability and reduces the ability to detect subtle forms of bias. Advances in optical design, calibration strategies and validation protocols that incorporate objective skin‐tone assessment will be essential to improve accuracy and ensure equitable performance across diverse patient populations.

## Funding

This work was supported in part by Coordenação de Aperfeiçoamento de Pessoal de Nível Superior (CAPES) under Grant 001, Fundação de Apoio ao Ensino, Pesquisa e Assistência do Hospital das Clínicas da Faculdade de Medicina de Ribeirão Preto and FAPESP under Grant 2017/25923‐5 and Grant 2013/07227‐0.

## Ethics Statement

The study was submitted to and approved by the Ethics Committee (CEP) of the University of São Paulo via Plataforma Brasil (CAAE 74692423.7.0000.5407, approved on 5 December 2023).

## Consent

All participants (or their legal surrogates) provided written informed consent prior to enrolment.

## Conflicts of Interest

The authors declare no conflicts of interest.

## Supporting information


**Data S1:** The completed GRRAS checklist supporting the reporting of this reliability and agreement study is available in the supplementary section.

## Data Availability

Data available upon request due to privacy/ethical restrictions.
