# Supplementary material for: Accuracy and Bias of Pulse Oximetry in the Intensive Care Unit: A Prospective Observational Study
Source: Nurs Crit Care. 2026 Mar 19;31(3):e70433. doi: 10.1111/nicc.70433 (PMC13002348; doi:10.1111/nicc.70433)
Supplement: Supplementary file 1 — Data S1: The completed GRRAS checklist supporting the reporting of this reliability and agreement study is available in the supplementary section. [file NICC-31-0-s001.doc]

**GRRAS checklist for reporting of studies of reliability and agreement – Pulse oximetry in ICU: accuracy and sin-tone bias**

| **Section** | **Item #** | **Checklist item** | **Reported on page #** |
| --- | --- | --- | --- |
| Title/Abstract | 1 | Identify in title or abstract that interrater/intrarater  reliability or agreement was investigated. | Page 3, Agreement (among three pulse oximeters) and Concordance (with arterial blood gas). |
| Introduction | 2 | Name and describe the diagnostic or measurement device of interest explicitly. | Page 6, Pulse oximetry. |
|  | 3 | Specify the subject population of interest. | Page 6-7, ICU patients. |
|  | 4 | Specify the rater population of interest (if applicable). | Page 6-7, Pulse oximeters in ICU settings. |
|  | 5 | Describe what is already known about reliability and  agreement and provide a rationale for the study (if applicable). | Page 6-7, Already reported low oximetry reliability in darker individuals and limited evidence with skin colorimetry and critically ill patients. |
| Methods | 6 | Explain how the sample size was chosen. State the determined number of raters, subjects/objects, and replicate observations. | Page 7-8, Samples chosen based on paired comparison of means. Three oximeters (raters) and arterial blood gas analysis, 100 patients and triplicate colorimetry are described. |
|  | 7 | Describe the sampling method. | Page 7-8, Data acquisition is described. |
|  | 8 | Describe the measurement/rating process (e.g. time interval between repeated measurements, availability  of clinical information, blinding). | Page 8-9, Processing and measurements are described. |
|  | 9 | State whether measurements/ratings were conducted independently. | Page 8, Measurements were obtained from three different pulse oximeters on the same subject, in immediate sequence. Arterial blood gas analysis was conducted independently as the reference. |
|  | 10 | Describe the statistical analysis. | Page 9, Methods contains Data Analysis section. |
| Methods/Results | 11 | State the actual number of raters and subjects/objects  which were included and the number of replicate observations which were conducted. | Pages 10, Results (table 1 and 2) include number of measurements (aprox. 100 per oximeter), oximeters (3) and subjects (100). |
| Methods/Results | 12 | Describe the sample characteristics of raters and  subjects (e.g. training, experience). | Pages 10, For oximeters used in Results, specifications were provided previously in Methods section, but others valuable information is displaced in the Results Tables. |
| Results | 13 | Report estimates of reliability and agreement including measures of statistical uncertainty. | Page 10-11, Figures 1-2 and Tables 2-3 provides bias, SD, ARMS, LoA, CCC, 95% CI and Deming Regression. |
| Discussion | 14 | Discuss the practical relevance of results. | Page 11-13, Considerations for use of oximeters in ICU and skin-tone/racial bias are described, impact and limitations also displaced. |
| Auxiliary material | 15 | Provide detailed results if possible (e.g. online). | The data that support the findings of this study will be made available upon request from the corresponding author. |

*As the checklist was provided upon initial submission, the page number/line number reported may be changed due to copyediting and may not be referable in the published version.
